# Research Progress Regarding Heat and Mass Transfer Characteristics of Agricultural Products Under Different Drying Methods, and Associated Applications: A Review

**DOI:** 10.3390/foods15142530

**Published:** 2026-07-17

**Authors:** Yue Yan, Tianhang Ding, Jiaoling Wang, Xuegeng Chen, Jikang Xu

**Affiliations:** 1 College of Mechanical and Electrical Engineering, Qingdao Agricultural University, Qingdao 266109, China; 13165470709@163.com (Y.Y.); chenxg130@sina.com (X.C.); 2 Nanjing Institute of Agricultural Mechanization, Ministry of Agriculture and Rural Affairs, Nanjing 210014, China; dingtianhang@caas.cn (T.D.); kclwjl@126.com (J.W.); 3 College of Mechanical and Electrical Engineering, Shihezi University, Shihezi 832003, China

**Keywords:** heat and mass transfer, porous media, drying technology, product quality, energy efficiency, carbon footprint, digital twin

## Abstract

Drying is a key operation for extending the shelf life of agricultural products and maintaining food quality, and its efficiency and product outcomes are governed by coupled heat and mass transfer. This review critically summarizes the mechanisms, technological characteristics, research methods and application prospects of agricultural-product drying from a heat- and mass-transfer perspective. The moisture-migration pathways, including surface evaporation, internal diffusion, capillary flow, vapor diffusion and bound-water desorption, are first discussed within a porous-medium framework. Governing equations based on Fourier’s law, Fick’s law, energy conservation and convective transfer are then introduced to clarify the theoretical basis of drying models. Typical convective, radiative, conductive and combined drying technologies are compared in terms of transfer mechanisms, drying efficiency, energy consumption, product-quality retention, carbon-footprint potential and industrial feasibility. Particular attention is given to the effects of drying-induced heat and mass transfer on color, texture, rehydration, bioactive compounds, antioxidant activity and microstructure. Current theoretical, experimental, numerical and data-driven research methods are further reviewed, and the limitations of existing studies are identified, including simplified homogeneous assumptions, insufficient model validation, limited quantitative comparison and weak scale-up applicability. Finally, future directions are proposed, including refined multi-scale and multi-field coupled models, advanced in situ characterization, multi-energy-field synergistic drying, digital twins, predictive modeling and multi-objective intelligent optimization. This review aims to provide a more mechanism-based and application-oriented reference for developing efficient, low-carbon and quality-preserving drying systems for agricultural products.

## 1. Introduction

Drying is one of the most important unit operations in the processing and storage of agricultural products, food materials, medicinal materials and biomass. By reducing moisture content and water activity, drying inhibits microbial growth, extends shelf life and improves transportation and processing adaptability. However, drying is not merely a dehydration process; it also directly affects color, texture, rehydration capacity, nutrient retention, flavor formation and the stability of bioactive compounds. Therefore, the development of efficient, energy-efficient and quality-preserving drying processes remains a central issue in food and agricultural-product processing engineering.

From a mechanistic perspective, agricultural-product drying is a transient and strongly coupled heat and mass transfer process. Heat is supplied to the material through convection, conduction and radiation to provide the latent heat required for water evaporation or sublimation. At the same time, internal moisture migrates toward the surface through liquid diffusion, vapor diffusion, capillary flow and pressure-driven flow, and is then removed by the surrounding drying medium [1,2]. As drying proceeds, the controlling mechanism generally shifts from external surface evaporation to internal diffusion resistance, which is often accompanied by shrinkage, microstructural collapse, surface hardening or local overheating [3].

A number of reviews have summarized drying kinetics, individual drying techniques and equipment development for agricultural products. Nevertheless, many existing reviews mainly describe technological categories and operating parameters, whereas fewer studies systematically compare different drying methods from the combined perspectives of transfer mechanisms, quantitative performance, product quality, energy consumption, carbon-footprint potential and industrial feasibility. In particular, the relationships among internal moisture migration, external heat supply, quality degradation and intelligent process control have not been sufficiently integrated into a unified review framework.

Compared with previous reviews, the novelty of this work lies in three aspects. First, this review uses heat and mass transfer as the central thread to compare convective, radiative, conductive and combined drying technologies rather than simply listing their working principles. Second, the discussion links transfer mechanisms with drying efficiency, effective moisture diffusivity, color retention, texture, bioactive compounds, energy efficiency, carbon footprint and industrial feasibility. Third, recent progress in numerical simulation, data-driven modeling, digital twins and predictive optimization is discussed to highlight future pathways toward intelligent and low-carbon drying systems. Overall, these features allow this review to move beyond a technology-by-technology description and to provide a more critical, mechanism-oriented and application-oriented comparison of drying technologies in terms of dominant transfer mechanisms, quality effects, energy performance, carbon-footprint potential and industrial feasibility.

## 2. Literature Landscape and Knowledge Gaps

Several recent reviews have summarized agricultural-product drying from the perspectives of drying kinetics, empirical and semi-empirical models, individual drying technologies, product-quality changes, low-carbon drying strategies and intelligent drying systems. These studies provide valuable references for understanding the application characteristics of different drying methods. However, many existing reviews focus mainly on a specific drying technology, drying-rate model or individual quality indicator, whereas the intrinsic relationships among heat supply, moisture migration, drying-stage evolution, quality changes, energy performance, carbon-footprint implications and industrial feasibility have not been sufficiently synthesized [4].

Compared with previous reviews, this review uses heat and mass transfer as the main analytical thread. It does not simply describe hot-air, microwave, infrared, vacuum or freeze drying separately; instead, it compares convective, radiative, conductive and combined drying technologies within a unified mechanism-oriented framework. Particular attention is paid to the dominant transfer pathway, rate-limiting step, transfer-enhancement mode, quality-related risk, energy-efficiency tendency, carbon-footprint potential and scale-up bottleneck of different drying technologies, thereby providing a more mechanism-based reference for efficient, low-carbon and quality-preserving agricultural-product drying systems.

From the perspective of transport mechanisms, porous-medium theory, Fickian diffusion models, effective moisture diffusivity, energy-balance equations, and multi-field coupled models remain the main tools for describing water migration and heat transfer in agricultural products. More recent modeling studies have extended traditional empirical and semi-empirical models toward physics-based numerical simulations and physics-informed data-driven models. These approaches are expected to improve the interpretability and predictive capability of drying models, especially for plant-based materials with heterogeneous cellular structures and complex pore networks [5].

In terms of drying technologies, conventional hot-air drying is still widely used because of its simple structure, stable operation, and high industrial adaptability. However, its heat and mass transfer processes are mainly governed by external convection and internal diffusion, which often leads to long drying time, high energy consumption, tissue shrinkage, surface hardening, and loss of heat-sensitive compounds. To overcome these limitations, various enhanced drying technologies, including solar drying, heat-pump drying, infrared drying, microwave drying, radio-frequency drying, vacuum drying, freeze drying, and combined drying, have been widely investigated. Physical-field-assisted drying technologies, such as microwave, radio-frequency, infrared, ultrasound, pulsed electric field, and high-voltage electric-field drying, can enhance heat and mass transfer by improving internal heating, reducing moisture migration resistance, or strengthening surface evaporation [6]. Nevertheless, these technologies may also cause uneven energy distribution, local overheating, equipment complexity, and difficulties in large-scale process control.

With the development of smart food processing and digital agriculture, online monitoring and intelligent optimization have become important research directions. Techniques such as near-infrared spectroscopy, hyperspectral imaging, computer vision, low-field nuclear magnetic resonance, dielectric-property measurement, and multi-sensor fusion have been applied to monitor moisture content, moisture distribution, shrinkage, color, and quality changes during drying [7,8]. In addition, artificial intelligence, machine learning, predictive control, and digital twins provide new possibilities for real-time optimization of drying processes. A digital twin-based drying system can integrate physical dryers, sensors, mathematical models, and optimization algorithms to dynamically predict temperature, moisture content, and quality attributes, thereby supporting intelligent decision-making and process control [9].

A comparison between Chinese and international studies shows both common interests and different research emphases. Studies in China have made substantial progress in the drying of fruits, vegetables, grains, Chinese medicinal materials, aquatic products, and other agricultural commodities. These studies often focus on drying kinetics, process-parameter optimization, equipment design, quality retention, and engineering application. In particular, many practical studies have been conducted on hot-air drying, heat-pump drying, infrared-hot-air drying, microwave-vacuum drying, radio-frequency-assisted drying, and combined drying systems. International studies also address drying kinetics and product quality, but recent work has placed more emphasis on multi-scale modeling, sustainability assessment, low-carbon drying, digital twins, advanced non-destructive monitoring, and life-cycle evaluation [4,9]. Therefore, future studies should not only compare drying methods based on drying time and final moisture content, but should also consider heat and mass transfer mechanisms, quality preservation, energy efficiency, carbon emissions, equipment cost, and industrial feasibility.

Despite these advances, several knowledge gaps remain. First, the relationship between microscopic moisture migration and macroscopic drying behavior is still insufficiently understood. Agricultural products are heterogeneous and anisotropic, and their cellular structure, pore distribution, and surface layers change dynamically during drying. However, many models still simplify agricultural products as homogeneous porous media with constant parameters, which limits their accuracy and general applicability. Second, the coupling between heat and mass transfer and product quality evolution has not been fully clarified. Many studies report the drying rate, effective moisture diffusivity, color, rehydration ratio, antioxidant activity, or nutrient retention separately, but few explain how local temperature gradients, moisture gradients, vapor pressure gradients, and structural changes jointly determine quality deterioration or retention.

Third, the industrial feasibility of emerging drying technologies remains insufficiently evaluated. Microwave drying, radio-frequency drying, infrared drying, vacuum drying, freeze drying, and multi-field combined drying can improve drying efficiency and product quality under certain conditions, but their equipment cost, control complexity, energy source, maintenance requirements, material adaptability, and large-scale drying uniformity are still major constraints. Fourth, low-carbon and sustainable drying has become an important but underdeveloped direction. Many studies still lack standardized indicators such as specific energy consumption, energy efficiency, exergy efficiency, greenhouse-gas emissions, and life-cycle carbon assessment [4]. Finally, intelligent drying systems are still at an early stage. Future research should develop physics-informed, data-driven, and sensor-integrated models that can simultaneously predict moisture content, temperature distribution, product quality, energy consumption, and carbon emissions. Such models will provide a theoretical and technical basis for high-efficiency, low-carbon, and quality-controlled drying of agricultural products.

Overall, these gaps indicate that the main challenge is not the absence of individual drying technologies but the insufficient integration of transport mechanisms, quality evolution and engineering applicability. Many laboratory studies have reported improved drying rates or quality retention under specific conditions, yet these advantages are often dependent on the material type, geometry, moisture content, thermal sensitivity and dryer structure, making direct scale-up difficult. For example, microwave and radio-frequency drying are constrained by dielectric properties, product thickness and field uniformity; vacuum and freeze drying are limited by equipment cost, energy demand and long processing cycles; and combined drying introduces multi-parameter coordination problems involving temperature, humidity, power, pressure and airflow. Therefore, future studies should move from single-index comparison toward multi-objective evaluation that simultaneously considers the drying rate, transfer uniformity, quality retention, energy consumption, carbon footprint and industrial feasibility.

## 3. Basic Theory of Heat and Mass Transfer During Agricultural-Product Drying

### 3.1. Mechanisms of Agricultural-Product Drying Process

Drying is a typical unsteady heat and mass transfer process in which heat input drives the migration of internal moisture and induces phase changes. On a macroscopic scale, the drying process can be divided into three stages: heating, constant-rate drying, and falling-rate drying. During the heating stage, the material temperature rapidly approaches that of the drying medium; however, the amount of moisture removed is relatively small. In the constant-rate stage, surface moisture evaporation dominates, and the drying rate is primarily governed by the external heat and mass transfer conditions. During the falling-rate stage, the free-water proportion gradually decreases while that of bound water increases, such that the resistance to internal moisture migration becomes the primary factor limiting the drying rate [10,11].

Owing to their porous structures, agricultural products exhibit complex internal moisture migration mechanisms, which typically involve capillary flow, water-vapor diffusion, and bound-water desorption. Capillary flow dominates the initial drying stages, whereas diffusion mechanisms become increasingly significant as the moisture content decreases. Thus, the overall drying process involves the combined action of multiple mass transfer mechanisms. Moreover, variations in cellular structure and pore characteristics among different agricultural products yield distinct drying kinetics, and this divergence poses a major challenge for the development of a unified drying model [10].

### 3.2. Theory of Heat Transfer During Drying

Heat transfer provides the energy basis for moisture evaporation and migration during the drying of agricultural products. The main modes include convection, radiation, and conduction. Under conventional hot-air drying conditions, convective heat transfer dominates, with heat being transferred from the drying medium through the boundary layer to the material surface and then into the interior of the material [12].

Heat conduction within a given material is typically described by Fourier’s law and is strongly influenced by the thermal conductivity, temperature gradients, and structural parameters. As moisture content changes continuously during drying, the effective thermal conductivity of agricultural products has pronounced non-linearity, which often necessitates empirical correction or experimental determination [13]. Under high-temperature or intensified drying conditions, the contribution of thermal radiation to the overall heat transfer becomes increasingly significant, particularly for infrared and combined drying methods [14]. Furthermore, heat transfer induces non-uniform internal temperature distributions, which can affect the material microstructure and quality; this aspect highlights the necessity of analyzing heat transfer in conjunction with moisture migration.

The basic conductive heat flux can be expressed by Fourier’s law:

***q*** = −*k*∇*T* (1)

where ***q*** is the heat flux, *k* is the thermal conductivity and ∇*T* is the temperature gradient. For an unsteady drying material, the heat balance can be written in a simplified form as:

*ρcp*(∂*T*/∂*t*) = ∇·(*k*∇*T*) + *Q* − *λ*(∂*M*/∂*t*) (2)

where *ρ* is density, *cp* is specific heat capacity, *Q* represents internal heat generation such as microwave or radio-frequency heating, λ is the latent heat of water evaporation and *M* is moisture content. This expression indicates that heat transfer and moisture removal are thermodynamically coupled through phase change.

### 3.3. Theory of Mass Transfer During Drying

For an agricultural product, the mass transfer behavior primarily governs water migration within the product and between the product and its surrounding environment. Internal water migration is typically dominated by diffusion and is often described using diffusion models based on Fick’s second law. The influences of material structure, temperature, and moisture content on water migration can be analyzed comprehensively by introducing an effective water diffusion coefficient [15].

For internal moisture diffusion, Fick’s second law is commonly used:

∂*M*/∂*t* = ∇·(*D*_eff_∇*M*) (3)

where *D*_eff_ is the effective moisture diffusivity. The temperature dependence of *D*_eff_ is often described using an Arrhenius-type equation:

*D*_eff_ = *D*_0_ exp[−*E_a_*/(*RT*)] (4)

where *D*_0_ is the pre-exponential factor, *E_a_* is the activation energy, *R* is the universal gas constant and *T* is absolute temperature. At the material surface, heat and moisture exchange with the drying medium can be simplified as *q*_c_ = *hA*(*T*_a_ − *T*_s_) and *N* = *kmA*(*C*_s_ − *C*_a_), where h and km are the convective heat and mass transfer coefficients, respectively. These equations provide the theoretical basis for evaluating the coupling between external transfer resistance and internal diffusion resistance.

The effective water diffusion coefficient varies with drying temperature and moisture content. Higher temperatures enhance the molecular motion, thereby increasing the diffusion rates and accelerating drying [16]. At the material surface, water is transferred to the drying medium via convective mass transfer, with the rate being determined by the mass transfer coefficient and water-vapor partial pressure difference [17]. During the falling stage of drying, the internal diffusion resistance becomes the primary factor controlling the overall drying rate. Therefore, accurate characterization of internal mass transfer is essential for modeling the drying kinetics.

### 3.4. Coupling Mechanisms and Mathematical Description of Heat and Mass Transfer

During drying, heat and mass transfer behaviors are tightly coupled through the phase change of water. Specifically, evaporation consumes latent heat and affects the internal temperature distribution of the material, and these temperature variations, in turn, influence the rate of water diffusion and the phase equilibrium state [18]. Consequently, the drying behavior cannot be accurately characterized by considering either the heat or mass transfer alone.

To address this complexity, coupled models based on the heat and mass conservation equations are commonly established to simultaneously resolve the temperature and moisture content fields. These models provide high-accuracy analysis of the drying kinetics and prediction of drying times; however, they require precise model parameters and substantial computational resources [19,20]. With advances in computational power, multi-field coupled numerical simulations have become essential tools for investigating heat and mass transfer behaviors during the drying of agricultural products.

## 4. Drying Technologies and Heat and Mass Transfer Characteristics

**Figure 1:**
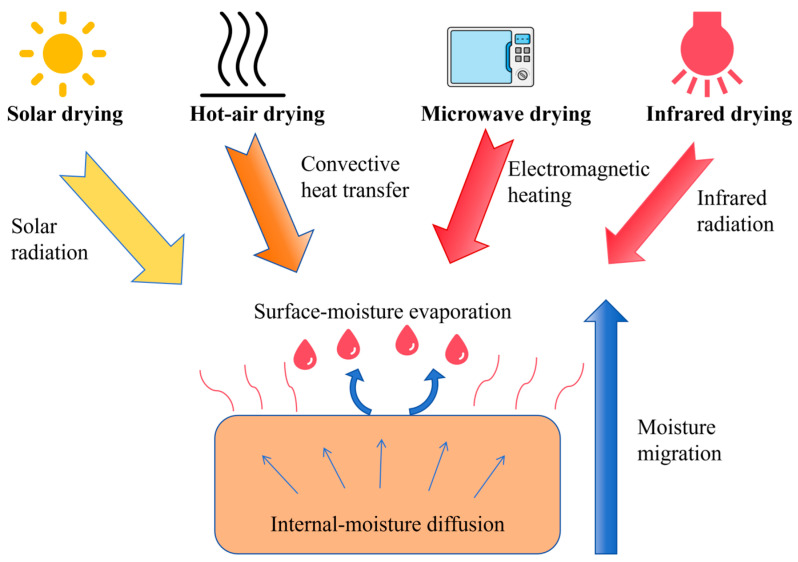
Schematic illustrating the heat and mass transfer mechanisms during the drying of an agricultural product.

The core principle of drying of agricultural products is the promotion of evaporation and outward diffusion of internal moisture through coupled heat and mass transfer. To achieve efficient drying, reduce energy consumption, and maintain product quality, the underlying heat and mass transfer mechanisms must be understood using various drying technologies. The heat and mass transfer behaviors associated with the various drying methods differ markedly, and directly influence the resultant drying rates, internal temperature distributions, and moisture migration pathways [21]. Figure 1 illustrates the fundamental heat and mass transfer mechanisms occurring during the drying of agricultural products, highlighting the interactions between the external heat supply, internal conduction and convection, and moisture movement.

### 4.1. Convective Drying Technologies

#### 4.1.1. Solar Drying

**Figure 2:**
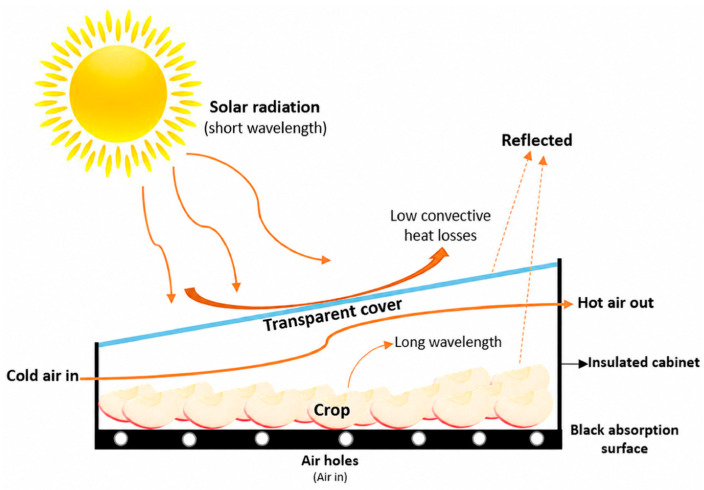
Schematic illustrating the solar-drying working principle [23].

Solar drying is an environmentally friendly method in which solar radiation acts as the primary heat source. Radiant energy is converted into thermal energy by collectors, drying-chamber structures, and auxiliary ventilation, thereby increasing the material temperature and promoting moisture evaporation. The energy input occurs primarily via radiative heat transfer, which is supplemented by natural or forced convective heat transfer. As the material surface absorbs this radiation, its temperature increases, and heat is conducted inward. Driven by the vapor pressure difference and concentration gradient, moisture migrates from the interior to the surface, where it evaporates into the surrounding air [22]. Figure 2 illustrates the working principle of solar drying and shows the interactions between the solar radiation and heat transfer within the material.

In recent years, extensive experimental and analytical studies have been conducted on the heat and mass transfer characteristics and structural optimization of solar-drying processes. For example, Kumar et al. [24] investigated the solar-drying behavior of various agricultural products. Those researchers noted that solar radiation intensity directly determines the surface temperature rise, thereby affecting the initial surface evaporation rate, whereas air velocity primarily influences the convective heat transfer coefficient and surface mass transfer capacity. Kidane et al. [25] evaluated the solar drying of Jinshuai apple slices under various loading conditions. By examining the effects of drying-chamber capacity on drying rates and effective moisture diffusion coefficients, they found that full-load conditions yielded higher drying efficiency, with the drying curves being dominated by a “deceleration phase.” These findings indicate that airflow and convective heat transfer significantly affect the heat and mass transfer behaviors. From the perspective of drying kinetics, solar drying typically yields a high rate of moisture evaporation during the initial stage, with internal moisture diffusion becoming the dominant rate-limiting factor during the middle and later stages. Experimental evidence supports this conclusion; for instance, for the solar drying of apple slices, a non-constant-rate phase dominates the process, with the moisture migration being strongly influenced by the internal diffusion resistance.

Structural optimization strategies involving heat collection panels and forced ventilation have been proposed as a means of addressing the unstable heat and mass transfer conditions that arise for traditional direct sun drying. For example, Kidane et al. [25] demonstrated that an appropriately designed drying chamber, along with controlled airflow within the dryer, partially mitigated the reduction in heat transfer intensity caused by uneven air convection. Hence, the drying uniformity and overall performance were improved. Furthermore, combining thermal energy storage materials or auxiliary energy sources with solar-drying systems can yield relatively stable heat and mass transfer behaviors under low-radiation or overcast conditions, thereby enhancing the drying efficiency and product quality. These findings provide practical guidance for the engineering of solar-drying equipment [26].

Overall, solar drying has advantages such as low commercial-energy dependence, low operating cost, environmental friendliness and reduced direct carbon-emission potential. However, its heat input and mass-transfer driving force are strongly affected by solar radiation intensity, ambient humidity and airflow fluctuation, which results in unstable drying efficiency, limited process controllability and variable product quality. Therefore, solar drying is more suitable for low-cost, small-scale or primary processing applications. For high-quality and continuous industrial production, it should be coupled with auxiliary heating, thermal-energy storage, hot-air drying or heat-pump systems to stabilize the drying environment and improve heat and mass transfer performance.

#### 4.1.2. Hot-Air Drying

**Figure 3:**
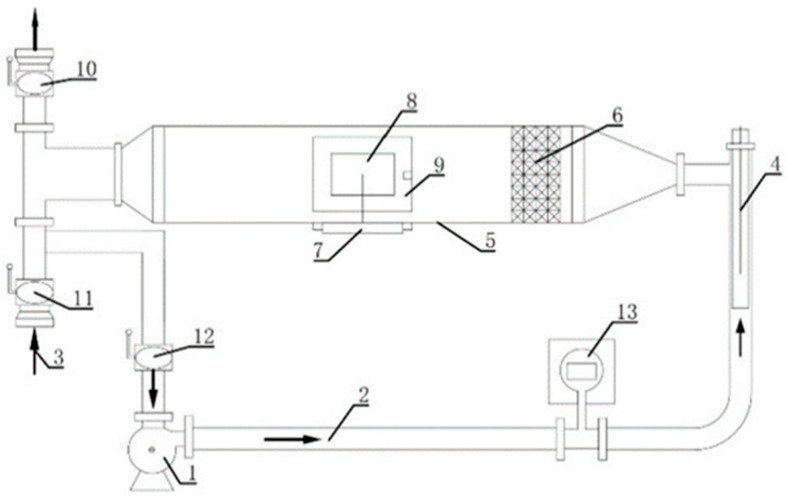
Schematic of the hot-air drying unit designed by Chen et al. [28]. 1. Blower; 2. piping; 3. air inlet; 4. heater; 5. drying chamber; 6. air distributor; 7. load cell; 8. material; 9. feed and discharge hatches; 10–12. butterfly valves; 13. gas flow meter.

For the drying of agricultural products, hot-air drying is currently the most widely applied technique. Its fundamental principle is that heated air makes direct contact with the target material, such that convective heat transfer supplies the latent heat required for moisture evaporation, and the generated water vapor is promptly removed. Heat is transferred from the air to the material surface through the boundary layer and conducted inward, whereas the internal moisture, driven by the vapor pressure difference and concentration gradient, migrates to the surface and undergoes a phase change. This constitutes a typical coupled process involving convective heat transfer and internal diffusion [27]. Figure 3 is a schematic of a hot-air drying apparatus designed by Chen et al. [28]; the configuration of the heating and airflow systems is illustrated.

Extensive research has demonstrated that agricultural products generally undergo three sequential stages under hot-air drying: heating, constant-rate drying, and falling-rate drying. During the constant-rate stage, surface-bound free-water evaporation dominates, and the drying rate is largely governed by external convective heat and mass transfer. In the falling-rate stage, the surface moisture decreases, the drying front moves inward, and the internal-moisture migration resistance increases, such that the process is predominantly controlled by internal diffusion [29]. To address the challenges associated with the falling-rate stage and the variability of the effective moisture-diffusion coefficient (*D*_eff_), Ju et al. [30] investigated the hot-air drying of carrot slices. Their study systematically examined the effects of the relative humidity and stage-specific dehumidification on the drying characteristics and transport processes. *D*_eff_ was estimated through model fitting, with rate-limiting behavior being observed in the later stages. Further, *D*_eff_ remained on the order of 10^−9^ m^2^/s under different humidity strategies, with progressive migration limitations arising as drying progressed.

Within an appropriate temperature range, hot-air drying can effectively preserve the basic structure and flavor characteristics of agricultural products. However, elevated temperatures may degrade heat-sensitive components such as vitamins and phenolic compounds, and may also induce tissue shrinkage and surface hardening [31]. Methods to enhance the heat and mass transfer behaviors and optimize the drying process have been investigated as means of mitigating the problems of high energy consumption and quality deterioration. In particular, segmented or variable-temperature drying strategies can maintain high drying rates in the early stages while reducing quality loss in later stages. For example, Xu et al. [32] reported that variable-temperature hot-air drying of shiitake mushrooms improved their color and rehydration capacity while reducing the total drying time compared with constant-temperature drying. Additionally, the combination of hot-air drying with other energy-field technologies, such as microwave, infrared, or heat-pump drying, can improve the internal heat and mass transfer behaviors, enhance the drying efficiency, and reduce the energy consumption.

In summary, hot-air drying remains one of the most mature and widely used technologies because of its simple equipment, stable operation, controllability and broad material adaptability. However, its transfer process is mainly governed by external convective heating, surface evaporation and later-stage internal diffusion. Once drying enters the falling-rate period, internal moisture diffusion becomes the dominant resistance, resulting in prolonged drying time, high specific energy consumption and possible quality deterioration, such as tissue shrinkage, surface hardening, color degradation and loss of heat-sensitive compounds. Therefore, optimization of hot-air drying should not rely only on increasing temperature, but should emphasize staged temperature–humidity control, airflow organization, exhaust-heat recovery and coupling with complementary technologies such as microwave, infrared or heat-pump drying.

#### 4.1.3. Belt Drying

**Figure 4:**
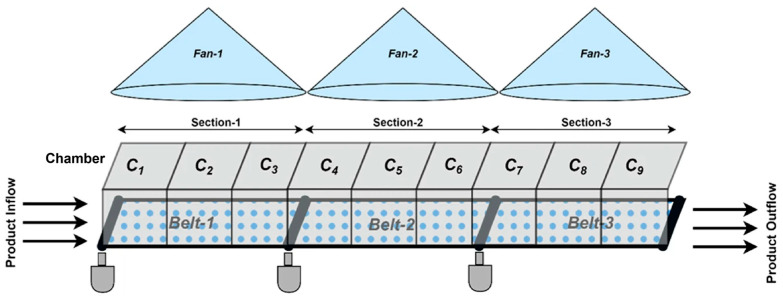
Schematic of conveyor belt dryer designed by Waseem et al. [33].

Belt drying is a typical continuous convective drying method in which the material is spread evenly in a thin layer on a conveyor belt and dehydrated in stages along the conveying direction within a multi-temperature-zone hot-air environment [10]. The primary heat transfer mode is forced convective heat exchange between the hot air and the material surface, which is accompanied by internal heat conduction. Moisture migration occurs as a coupled mass transfer process involving surface evaporation and internal diffusion. Although the mechanism is similar to that of a hot-air chamber dryer, the thin-layer distribution significantly shortens the heat conduction path and moisture diffusion distance, thereby enhancing the overall heat and mass transfer. Figure 4 is a schematic of a conveyor belt dryer designed by Waseem et al. [33].

In continuous belt-drying processes, the organization and uniformity of the airflow field directly influence the convective heat transfer intensity and surface mass transfer capacity. Specifically, an uneven airflow distribution increases the differences in the temperature and humidity boundary layer surrounding the material at different locations, which can easily yield non-uniform drying. Gong et al. [34] conducted CFD-based numerical simulations and structural optimization studies on a multi-temperature-zone mesh belt dryer, taking the temperature and flow-field distribution characteristics as evaluation criteria. Their results indicated that optimizing the airflow distribution and structural parameters improved the temperature and flow-field uniformity across different zones; hence, they established a design basis for enhancing the heat and mass transfer consistency in mesh belt drying. Similarly, Dong et al. [35] established a porous-medium model and performed numerical flow-field simulations on a mesh-belt-type Sichuan pepper dryer to investigate the influence of the baffle parameters on the airflow intensity and uniformity. Their findings suggest that a rational baffle configuration can improve the airflow distribution within the drying chamber, thereby reducing the drying non-uniformity and contributing to stable product quality.

To address the high energy consumption of belt dryers, researchers have increasingly integrated energy-saving technologies and methods to enhance the heat and mass transfer performance. The introduction of heat pumps and waste-heat recovery has improved the energy efficiency of these systems. Additionally, the development of hybrid approaches combining belt drying with microwave or infrared drying has enabled rapid initial-stage dehydration followed by later-stage uniform convective drying, with the total drying time being reduced and the product quality being improved.

Overall, belt drying offers continuous operation, high automation, large processing capacity and good industrial scalability. Nevertheless, it remains a convective drying process in which later-stage dehydration is limited by internal moisture migration. Material-layer thickness, local airflow distribution and zone-temperature design strongly influence temperature and moisture gradients during continuous production and may lead to non-uniform drying. Therefore, CFD-assisted airflow optimization, rational zonal temperature–humidity control, online moisture monitoring, waste-heat recovery and integration with microwave or infrared-assisted drying are important for improving transfer uniformity, reducing energy loss and stabilizing product quality in belt dryers.

#### 4.1.4. Desiccant-Wheel-Assisted Convective Drying

**Figure 5:**
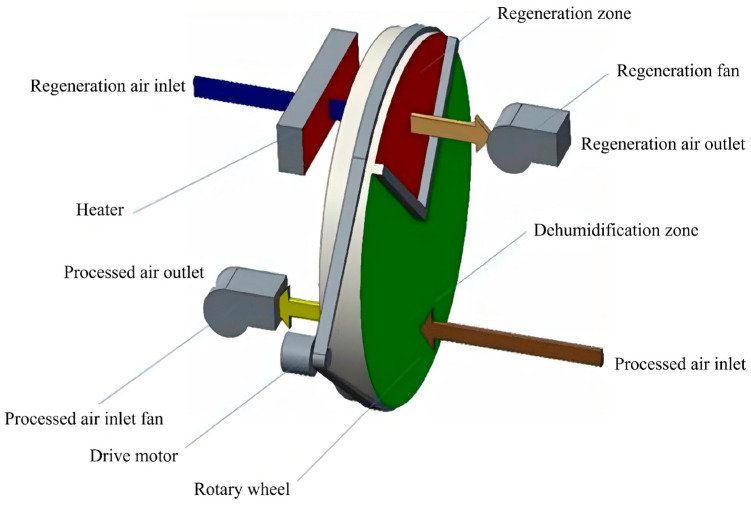
Schematic of a desiccant-wheel structure [37].

Desiccant-wheel-assisted convective drying improves the moisture-evaporation driving force by controlling the humidity of the drying air. During convective drying, the air humidity critically impacts the moisture-removal rate. Lowering the absolute humidity of the air significantly increases the water-vapor partial pressure difference between the material surface and the surrounding air, thereby intensifying the drying process. Figure 5 is a schematic of a desiccant wheel. Desiccant-wheel technology typically employs a wheel coated with hygroscopic materials, such as silica gel or molecular sieves, to adsorb water vapor from the air and, thus, produce low-humidity air. The dehumidified air is subsequently reheated and fed into the drying chamber, where it interacts with the material to promote moisture evaporation and migration [36].

Desiccant-wheel drying is a typical convective drying process centered on heat and mass transfer. In detail, heat is primarily delivered to the material via convective heat exchange at the surface and then conducted inward, thereby supplying the latent heat required for moisture evaporation. Simultaneously, the internal moisture migrates toward the surface under vapor pressure and concentration gradients, and is removed by the airflow [38]. Because dehumidified air has a lower dew point and reduced relative humidity, the mass transfer driving force between the drying air and material is substantially higher than that obtained in conventional hot-air drying. This feature enables the maintenance of high drying rates at lower temperatures and offers distinct advantages for the drying of heat-sensitive agricultural products and foods.

Recent studies have extensively investigated the effects of the air state parameters and operating conditions on the drying performance of desiccant wheel systems. Air-humidity reduction has been identified as a key factor facilitating improved drying rates while also influencing the system energy efficiency. For example, in low-temperature peanut drying, a combined desiccant wheel and heat-pump system was found to significantly lower the inlet-air moisture content, accelerate the moisture migration, and reduce the drying time [39]. Similarly, low-temperature, low-humidity air was shown to enhance the drying rate and improve the color and texture of dried fish [40]. Research has further demonstrated that desiccant-wheel technology can stabilize the drying-air dew point, thereby improving the process controllability and reducing the loss of heat-sensitive components [36].

To further improve the energy efficiency, desiccant dehumidification has been increasingly integrated with heat-pump systems, thereby enabling energy recovery through latent and sensible heat recycling. Such hybrid systems maintain low-humidity environments and provide stable thermal energy, significantly improving the coefficient of performance (COP) and reducing the energy consumption per unit product [39]. These systems have been successfully applied to the drying of fruits, vegetables, seafood, and biomass, and have exhibited both energy-saving potential and product-quality preservation.

In summary, desiccant-wheel-assisted convective drying enhances the mass-transfer driving force by reducing the humidity and dew point of the drying air and increasing the vapor-pressure difference between the material surface and the surrounding medium. This enables efficient low-temperature drying and is particularly suitable for heat-sensitive agricultural products and foods. However, compared with conventional hot-air drying, the system is structurally more complex and usually requires additional regeneration heat and higher equipment investment. Its industrial advantage therefore depends on reducing regeneration energy, improving heat-recovery efficiency and rationally integrating the desiccant wheel with heat-pump or renewable-energy systems.

### 4.2. Radiative Drying Technologies

Depending on the energy transfer mode, radiative drying can be classified into electromagnetic-field drying (such as microwave (MW) drying and radio-frequency (RF) drying) and surface radiation drying (such as infrared radiation (IR) drying).

#### 4.2.1. Electromagnetic-Field Drying (MW and RF Drying)

The electromagnetic-field drying technique utilizes electromagnetic energy to generate heat directly within a material, thereby accelerating moisture evaporation and migration. Typical methods include MW and RF drying. Unlike conventional hot-air drying, which primarily involves convective heat transfer, electromagnetic-field drying delivers energy volumetrically, thereby shortening the heat conduction path and establishing a substantial internal vapor-pressure gradient that drives rapid moisture migration toward the surface [41].

**Figure 6:**
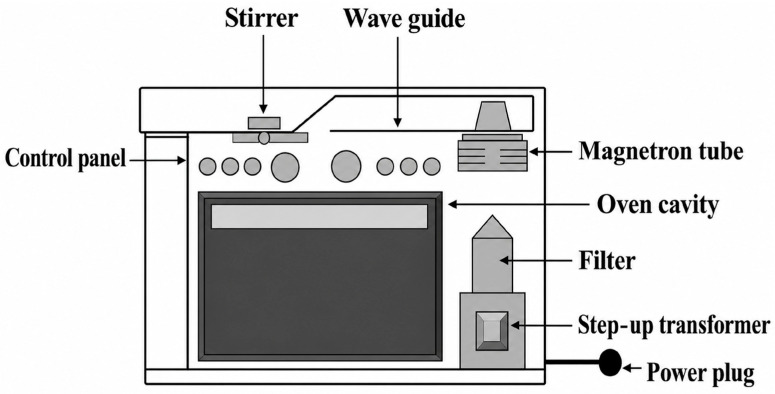
Schematic of an MW drying unit [45].

MW drying typically employs high-frequency electromagnetic fields of 915 or 2450 MHz. Thermal energy is generated through the vibrational friction of polar water molecules in an alternating electric field; this energy then heats the material from the inside. From the perspective of heat and mass transfer, the energy is primarily produced endogenously, which significantly reduces the conduction path. The moisture migration is driven not only by concentration gradients but also by internal vapor-pressure gradients, such that the diffusion- and pressure-driven mechanisms are coupled [42]. Zhu et al. [43] investigated water migration during the MW drying of agricultural products using thermogravimetric–NMR technology. They demonstrated that MW treatment accelerated the conversion of free water to bound water and enhanced the internal-moisture migration rate toward the surface, thereby improving the overall drying efficiency. Zhao et al. [44] further noted that MW heating can be uneven in thick materials, such that significant thermal and moisture gradients are produced; this may compromise the drying uniformity. Figure 6 is a schematic of an MW drying apparatus.

**Figure 7:**
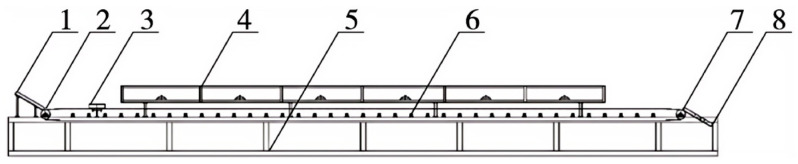
Overall structure diagram of an RF dryer [49]. 1. Feed inlet; 2. conveyor belt; 3. thickness-limiting device; 4. RF box; 5. bottom bracket; 6. electric heating device; 7. roller wheel; 8. discharge port.

Compared with MW drying, RF drying operates at lower frequencies, typically 13.56 or 27.12 MHz, and offers greater penetration depth. Thus, RF drying enables the simultaneous heating of thick layers or large-volume materials, thereby improving the drying uniformity and mitigating hot-spot effects [46]. RF drying is a typical volumetric heating process in which the heat generated internally establishes a vapor-pressure gradient that drives moisture from the interior to the surface, while convective heat transfer at the material surface facilitates moisture removal [47]. This mechanism yields high efficiency and uniformity for RF drying, particularly when applied to thick-layered agricultural products, vegetables, and fruits. Experimental studies have demonstrated that RF drying not only accelerates the process but also preserves the colors and textures of heat-sensitive materials under low-temperature conditions. For instance, in apricot-drying experiments, Topcam et al. [48] reported that RF heating significantly reduced the drying time, minimized the temperature gradient between the surface and interior, and enhanced the moisture migration uniformity, thereby improving the product quality. Additionally, RF drying of thick-walled fruits, such as goji berries, has been shown to maintain uniform internal temperatures, which favor the retention of active compounds and antioxidants [46]. Figure 7 shows the overall structure of an RF dryer.

Overall, both MW and RF drying intensify internal heat generation and moisture migration through volumetric electromagnetic heating, but they differ in penetration depth, heating uniformity and material suitability. MW drying is more appropriate for thin-layered or small-to-medium-sized products, whereas RF drying, with its larger penetration depth, is more suitable for thick-layered or large-volume materials. Despite their high drying efficiency, non-uniform electromagnetic-field distribution, material-dependent dielectric properties and hot-spot formation remain key bottlenecks affecting transfer uniformity and product quality. Industrial application therefore requires optimized cavity or electrode design, appropriate power or frequency adjustment, real-time process monitoring and integration with convective moisture removal or other complementary drying methods.

#### 4.2.2. IR Drying

**Figure 8:**
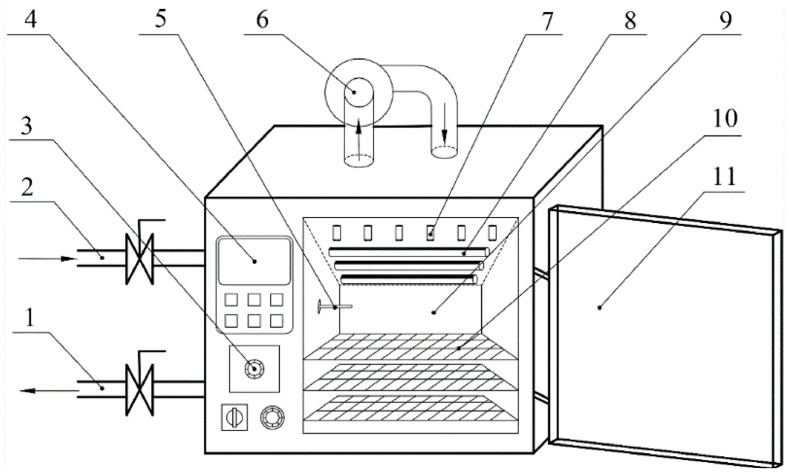
Schematic of an IR dryer [53]. 1. Wet discharging port; 2. air inlet port; 3. wind speed adjusting knob; 4. touch screen; 5. temperature sensor; 6. centrifugal fan; 7. spray nozzle; 8. quartz infrared heating tube; 9. drying chamber; 10. material tray; 11. door.

IR drying is a radiant heating method in which an IR source emits energy onto the material surface. The material absorbs this radiation and converts it into thermal energy, thereby promoting moisture evaporation [50]. During IR drying, heat is primarily transferred to the surface layer through radiation and subsequently conducted into the interior. Moisture migration results from the synergistic action of rapid surface evaporation and internal diffusion. Thus, IR drying is characterized by enhanced surface heat transfer [51]. From the perspective of heat and mass transfer, IR rapidly increases the surface temperature within a short period, significantly increasing the surface-moisture evaporation rate and creating a steep surface-temperature gradient, thereby strengthening the driving force for internal moisture diffusion [52]. Compared with conventional hot-air drying, IR drying yields higher heating rates and greater initial-stage heat and mass transfer intensities, which reduce the overall drying time. Figure 8 is a schematic of a typical IR dryer.

To investigate the effects of process parameters on heat and mass transfer behaviors during IR drying, Lin et al. [54] studied combined IR–hot-air drying of white radish slices. Using numerical simulations and drying experiments, they analyzed the influence of the radiation power and drying stage on the temperature and humidity fields. The results indicated that increasing the IR intensity accelerated surface heating and moisture migration. However, excessive radiation intensity increased the risk of localized overheating; this phenomenon could damage tissue structures and degrade product quality. Delfiya et al. [55] investigated the effects of material thickness on the combined IR–hot-air drying of carrot slices. They found that thinner materials exhibited more uniform IR energy absorption, yielding more favorable heat and mass transfer and improved drying uniformity, whereas thicker materials exhibited pronounced thermal and moisture gradients, which impeded uniform moisture release.

As IR has a limited penetration depth, IR drying has been combined with convective hot-air drying. In these combined methods, IR is applied in the initial stage for rapid surface dehydration, with hot-air convection being employed in the later stage to facilitate internal moisture migration and uniform drying. Jiang et al. [56] reported that combined IR–hot-air drying of white radish slices reduced the drying time by more than 25%, with the effective moisture diffusion coefficient being significantly increased compared with hot-air drying alone. Similar improvements in drying performance have been observed for shiitake mushrooms and yam slices [57,58].

In summary, IR drying accelerates heat and mass transfer mainly by rapid surface radiative heating, which increases the initial surface-evaporation rate. It is particularly suitable for thin-layer materials or early-stage dehydration. However, because of its limited penetration depth, IR drying may form large surface-to-interior temperature and moisture gradients in thick or irregular materials, leading to surface overheating, insufficient internal moisture migration and non-uniform drying. Therefore, compared with single IR drying, combined methods such as IR–hot-air or IR–vacuum drying are more promising for balancing drying efficiency, transfer uniformity and quality retention.

### 4.3. Conductive Drying Technologies

#### 4.3.1. Vacuum Drying

**Figure 9:**
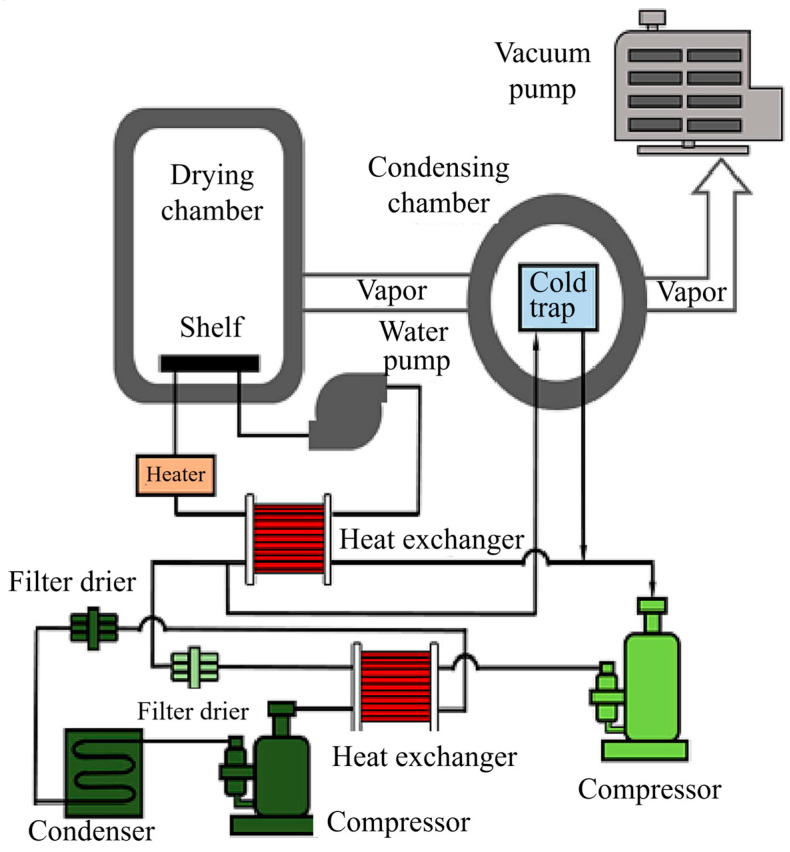
Schematic of a vacuum-drying device [61].

Vacuum drying is a low-temperature dehydration method employing reduced pressure within the drying chamber such that the boiling point of water is lowered significantly. Compared with atmospheric drying, the reduced boiling point obtained under vacuum conditions facilitates moisture removal at lower temperatures, effectively mitigating the thermal degradation of heat-sensitive components [59]. The vacuum environment increases the vapor pressure difference between the interior and exterior of the material, thereby enhancing the water-evaporation driving force. Simultaneously, the lower drying temperature slows the rate of heat conduction within the material, such that the heat transfer is a potential limiting factor. Therefore, during vacuum drying, the heating method and intensity must often be optimized to ensure a continuous supply of latent heat for moisture evaporation [60]. Figure 9 is a schematic of a rack-type vacuum dryer.

Recent studies have focused on the drying behavior and process characteristics arising under vacuum conditions. For fruits and vegetables, Ren et al. [62] compared vacuum drying with hot-air drying and demonstrated that the former facilitates moisture vaporization at lower temperatures, shortens overall drying time, and improves drying efficiency. Notably, the underlying heat and mass transfer mechanisms of these methods differ significantly, indicating that the vacuum conditions alter both the drying driving force and moisture migration patterns. Dai et al. [63] investigated the drying characteristics of red-stemmed bamboo fungi using various drying methods and found that vacuum infrared drying yielded a faster initial dehydration rate and higher overall drying efficiency than conventional hot-air drying. This improvement was attributed to the reduced vapor pressure obtained under vacuum, which strengthened the vaporization driving force and altered the heat and mass transfer dynamics. These findings highlight the fundamental difference in the mass transfer behavior under vacuum conditions compared to that under atmospheric drying, with moisture vaporization occurring more readily during the vacuum stage.

To further enhance the drying performance, vacuum environments are often combined with other energy-field technologies. For instance, MW–vacuum drying, which couples volumetric MW heating with low-pressure evaporation, can significantly reduce drying time while improving product quality. Zhang et al. [64] reviewed the use of MW–vacuum drying for fruits, vegetables, and medicinal herbs and noted its high drying efficiency and excellent quality retention. Similarly, those researchers used IR–vacuum combined drying to increase surface heating rates and improve overall heat transfer conditions.

In summary, vacuum drying modifies the phase-change condition of water by reducing the ambient pressure, thereby enhancing moisture evaporation at relatively low temperatures and reducing thermal and oxidative damage. This makes it suitable for heat-sensitive and high-value agricultural products. However, convective heat transfer is weakened under low-pressure conditions, and heat supply may become the rate-limiting step. In addition, vacuum dryers usually require high sealing performance, complex structure and relatively high capital and operating costs. Therefore, their industrial feasibility depends on whether the product-quality premium can offset the added equipment and energy costs, while future improvements should focus on heat-transfer enhancement, energy recovery and coupling with microwave or infrared heating.

#### 4.3.2. Freeze Drying

**Figure 10:**
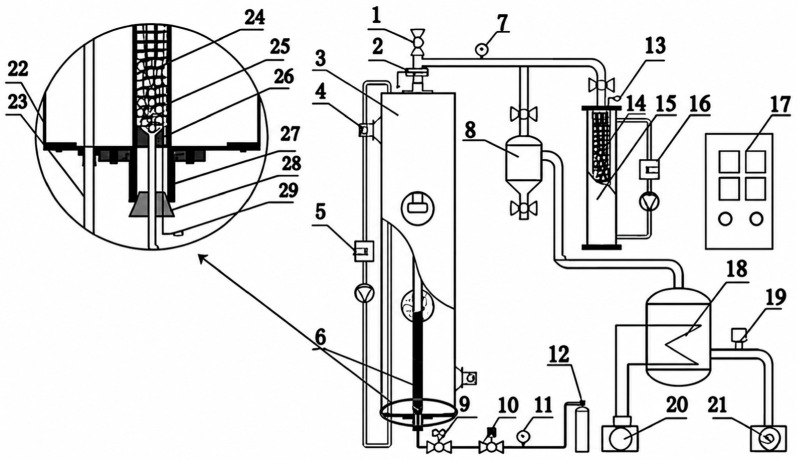
Schematic of a freeze-drying device [66]. 1. Feed-ball valve; 2. plate valve with 3 mm aperture; 3, 22. MW heating chamber; 4. magnetic tube; 5, 16. circulating water unit; 6. MFD and PSMFD drying chamber; 7, 11. pressure meter; 8. solid–gas separator; 9. gas-flow solenoid valve; 10. gas-flow regulating valve; 12. nitrogen source (7–12 for pulse-spouted microwave-assisted freeze drying only); 13, 29. optical-fiber temperature sensor; 14, 24. sample; 15. freeze drying chamber with jacket; 17. control panel; 18. steam condenser; 19. vacuum pressure sensor; 20. refrigeration unit; 21. vacuum pump unit; 23. water loading tube; 25. Teflon tube; 26. gas distributor; 27. drying-chamber bracket fixing device; 28, 29. silicone rubber plug (×4).

Freeze drying is a low-temperature dehydration method in which the material is first frozen, with the resulting ice crystals then being removed via direct sublimation under low-pressure conditions. The process generally comprises three stages: freezing, primary drying (sublimation), and secondary drying (desorption). Unlike conventional evaporative drying methods, freeze drying removes moisture primarily through solid-state sublimation; hence, structural damage caused by liquid-water migration or high-temperature exposure is avoided. Therefore, freeze drying is considered a typical low-temperature phase-change drying technology [65]. Figure 10 shows the schematic of a freeze-drying apparatus.

From the perspective of heat and mass transfer, the energy supplied during freeze drying is primarily used to compensate for the latent heat required for ice sublimation. Heat is typically conducted from the heating plate through the material to the sublimation interface. The water vapor diffuses outward along a pressure gradient through the porous drying layer and is removed by the vacuum system. As the sublimation front progresses, both the thermal conduction resistance and water-vapor diffusion resistance increase, causing a gradual decrease in the sublimation rate. Consequently, freeze drying is often characterized by the dual limitations of heat and mass transfer [67].

The mechanisms governing heat and mass transfer during the freeze-drying process have been extensively investigated, and methods to enhance these behaviors have been developed. Yao et al. [68] demonstrated that the size and distribution of the ice crystals that form during the freezing stage strongly influence the pore structure during sublimation, thereby affecting the water-vapor diffusion pathways and drying rates. Slower freezing generally promotes larger ice crystals and interconnected pores, which reduce the mass transfer resistance. Al Faruq et al. [66] noted that optimization of both the freezing protocol and heat supply strategy during sublimation can significantly reduce the drying time while maintaining product quality.

Various enhancement strategies have been proposed to overcome the intrinsic limitations of low heat and mass transfer efficiency and prolonged drying times. These methods include MW- and IR-assisted freeze drying, along with pulsed heating. MW-assisted freeze drying leverages volumetric heating to deliver energy directly to the material interior during the sublimation stage, thereby reducing the internal thermal resistance and shortening the drying time [69]. IR can also be employed to supplement the heat supply at the sublimation interface, thereby enhancing the overall energy transfer.

In summary, freeze drying achieves low-temperature dehydration through ice sublimation and can maximize the retention of morphology, pore structure, color, nutrients and bioactive compounds. It is suitable for high-quality fruits, vegetables, medicinal herbs, functional foods and other high-value products. However, as the sublimation front advances, the thermal conduction resistance of the dried layer and the water-vapor diffusion resistance gradually increase, leading to long processing cycles, high capital investment and high energy consumption. Therefore, freeze drying is more suitable for high-value products, and future work should focus on optimized freezing protocols, enhanced heat supply, microwave- or infrared-assisted freeze drying and multi-field coupling control to shorten drying time and reduce energy consumption.

### 4.4. Combined Drying

As the limitations of single-method drying in terms of efficiency, energy consumption, and product quality have become increasingly apparent, the integrated application of multiple drying technologies has emerged as a key strategy for enhancing drying performance. Combined drying involves the synergistic use of two or more drying methods, enabling simultaneous operation of multiple heat and mass transfer mechanisms within a single drying process. This approach can enhance the heat input and moisture migration behavior throughout the drying cycle, thereby improving drying efficiency, reducing energy consumption, and preserving product quality [70].

In typical combined drying processes, different methods are dominant at different stages. For instance, in MW–hot-air combined drying, microwave energy rapidly heats the interior of the material through volumetric heating, thereby creating a vapor pressure gradient that drives the moisture migration from the interior to the surface. Simultaneously, hot air removes the evaporated moisture via convective mass transfer, thereby lowering the surface water-vapor partial pressure and enhancing the overall mass transfer rate [71]. This combined approach can significantly shorten drying time and improve drying efficiency [72].

Similarly, in IR–hot-air combined drying, IR rapidly heats the surface layer of the material, thereby increasing the initial surface evaporation rate, whereas in the later stages, hot air promotes internal moisture migration, thereby ensuring a more uniform drying process [73]. In low-temperature drying applications, combined heat pump–dehumidification drying can reduce air humidity and enable thermal energy recovery, yielding drying air with a lower dew-point temperature. This property maintains a larger vapor pressure difference between the material and air, thereby enhancing the moisture-migration driving force [74].

Moreover, coupling electromagnetic-field technologies with low-pressure environments, such as MW–vacuum drying, can further improve the heat and mass transfer efficiency. The vacuum environment lowers the boiling point of water, whereas the MW volumetric heating intensifies the internal evaporation, yielding accelerated moisture removal [75]. From a mechanistic perspective, the main advantage of combined drying is the synergistic effect of “internal volumetric heating coupled with external convective mass transfer,” which optimizes the spatial distribution of the temperature and moisture content fields, reduces the internal diffusion resistance, and enhances the overall drying efficiency [76].

In summary, combined drying is not a simple superposition of two or more dryers, but a stage-matched strategy that coordinates energy input and moisture removal according to the dominant heat and mass transfer limitation at different drying stages. For example, MW–hot-air drying combines internal volumetric heating with external convective vapor removal; IR–hot-air drying strengthens early surface evaporation and later uniform dehydration; and MW–vacuum drying couples volumetric heating with low-pressure evaporation. Although combined drying can improve drying efficiency, reduce energy consumption and maintain product quality, it involves coordinated control of temperature, humidity, electromagnetic power, pressure, airflow and product state. Future applications should therefore emphasize real-time monitoring and multi-objective optimization to balance drying efficiency, energy use and quality retention.

### 4.5. Summary

This section systematically reviewed typical drying technologies and highlighted the heat and mass transfer characteristics of drying of agricultural products. To facilitate a comparative analysis of different drying methods, Table 1 summarizes their key aspects, including heat and mass transfer mechanisms and transfer characteristics.

From a comparative perspective, different drying technologies are limited by different dominant transfer resistances. Hot-air drying, belt drying and desiccant-wheel-assisted convective drying mainly rely on external convective heat transfer and surface moisture evaporation. These technologies have mature equipment structures and strong industrial adaptability, but in the middle and late drying stages they are often limited by internal moisture diffusion, leading to a decreased drying rate, increased energy consumption and possible shrinkage, hardening or loss of heat-sensitive compounds. Therefore, their improvement should focus not only on increasing temperature, but also on airflow distribution, air dehumidification, internal moisture migration enhancement, exhaust-heat recovery and drying uniformity.

Radiative and electromagnetic-field drying technologies, including MW, RF and IR drying, can intensify heat and mass transfer through surface radiation heating or internal volumetric heating. These methods can shorten drying time, improve the initial drying rate and strengthen the driving force for moisture migration. However, their practical performance depends strongly on product thickness, dielectric or absorption properties and energy-field distribution. Improper control may cause local overheating, non-uniform drying and quality damage. Therefore, industrial application should consider not only drying efficiency but also field uniformity, equipment structure, online monitoring and matching with convective moisture removal.

Vacuum and freeze drying reduce thermal damage by modifying pressure and phase-change conditions, making them suitable for heat-sensitive, high-quality or high-value agricultural products. Nevertheless, their scale-up is constrained by heat-transfer limitations, equipment cost, energy consumption and long processing cycles. In contrast, combined drying should be regarded as a mechanism-matching strategy rather than a simple equipment combination, because it coordinates energy input and moisture removal according to the dominant transfer limitation in each drying stage. Therefore, drying technology selection should shift from single-efficiency judgment to comprehensive matching among material properties, transfer mechanisms, quality targets, energy cost and industrial feasibility.

In addition to heat and mass transfer characteristics, the practical application of drying technologies also depends on industrial feasibility, energy efficiency and carbon-footprint performance. A drying method with high transfer efficiency may not always be suitable for large-scale application if it requires high equipment investment, complex process control or excessive energy input. Therefore, a comprehensive evaluation should consider not only the drying rate and product quality, but also equipment maturity, scalability, energy source, heat recovery potential and carbon-emission reduction. Table 2 summarizes the industrial feasibility, energy-efficiency tendency, carbon-footprint considerations and main bottlenecks of different drying technologies.

As shown in Table 2, hot-air and belt drying have the highest industrial maturity and scalability, but their relatively long drying time and heat loss may result in high specific energy consumption. Solar drying has the greatest potential for reducing direct carbon emissions, but its instability limits its use in high-quality industrial production. Desiccant-wheel drying can improve energy efficiency through humidity control and heat recovery, whereas MW, RF and IR drying can shorten drying time by intensifying heat and mass transfer, although their carbon-footprint performance depends strongly on electricity sources and process uniformity. Vacuum and freeze drying are more suitable for high-value products because of their quality-preserving advantages, but their high equipment cost and energy demand restrict large-scale application. In comparison, combined drying provides a promising route for balancing drying efficiency, product quality and energy consumption, but requires more advanced multi-parameter control and optimization.

## 5. Effects of Drying-Induced Heat and Mass Transfer on Product Quality

The value of a drying technology should be evaluated not only by the moisture removal rate but also by its effects on product quality. Heat and mass transfer determine the thermal load, moisture gradients and structural stresses experienced by agricultural products, and these factors directly affect color, texture, rehydration, bioactive compounds, antioxidant activity and flavor stability [77]. Therefore, quality changes should be interpreted together with transport mechanisms rather than treated as independent evaluation indices.

### 5.1. Color, Browning and Appearance

Color degradation during drying is closely related to surface temperature, oxygen exposure, moisture content and drying duration. Conventional hot-air drying may intensify enzymatic or non-enzymatic browning when products remain at moderate-to-high temperatures for a long period [78]. IR and MW drying can shorten processing time, but excessive local heating may accelerate pigment degradation or surface browning [79]. Vacuum and freeze drying reduce oxygen exposure and thermal damage, thereby generally improving color retention for heat-sensitive materials. Quantitative indicators such as color difference (ΔE), browning index and pigment retention should therefore be considered together with drying kinetics.

### 5.2. Texture, Shrinkage and Rehydration

Moisture gradients and internal pressure changes can induce shrinkage, collapse, cracking or surface hardening. Slow convective drying often causes large internal diffusion resistance and prolonged structural stress, whereas MW and RF heating may create rapid internal vapor pressure that accelerates moisture migration but may also damage cellular structures if not properly controlled [80]. Freeze drying maintains an open porous structure through ice sublimation, which improves rehydration capacity but at the expense of long processing time and high energy consumption [81]. Thus, texture and rehydration properties reflect the balance between dehydration intensity and microstructural preservation.

### 5.3. Bioactive Compounds, Antioxidant Activity and Nutrient Preservation

Bioactive compounds, including phenolics, flavonoids, vitamins, pigments and volatile components, are sensitive to temperature, oxygen, moisture state and drying duration [82]. Short-time intensified drying may reduce thermal exposure, whereas excessive electromagnetic or radiative energy can cause local overheating and nutrient degradation [83]. Low-temperature vacuum and freeze drying usually provide better retention of thermolabile nutrients and antioxidant activity, but their cost and energy demand restrict large-scale application [84]. For this reason, future comparisons should include not only drying time and *D*_eff_ but also retention rates of representative nutrients, antioxidant capacity, flavor compounds and specific energy consumption.

### 5.4. Microstructure–Quality–Transfer Relationship

Advanced characterization techniques such as NMR, CT, MRI, infrared thermography and scanning electron microscopy can reveal water-state transformation, pore evolution, tissue collapse and temperature distribution during drying [85]. These observations help link microscopic moisture migration with macroscopic quality outcomes. A mechanism-based quality evaluation framework should therefore combine drying kinetics, internal moisture distribution, thermal history, microstructure evolution and final quality indices [86]. Such integration is especially important for developing digital twins and predictive quality-control models for industrial drying systems.

## 6. Research Methods for Analysis of Heat and Mass Transfer Mechanisms

The heat and mass transfer behaviors occurring during the drying of agricultural products are inherently complex and influenced by multiple factors, including the material structure, drying method, and process parameters. To improve the drying efficiency and product quality, these mechanisms must be understood, the drying kinetics must be accurately predicted, and the process conditions must be optimized. To achieve these objectives, extensive investigations have been conducted using a combination of theoretical modeling, experimental research, and numerical simulations.

This section presents a systematic summary and analysis of the main research methods employed to study the heat and mass transfer behaviors occurring during the drying of agricultural products: their principles, applications, advantages, and limitations. By elucidating the strengths and scope of each approach, a methodological foundation is established to enable future studies on drying-process optimization and to further the development of high-performance drying technologies.

### 6.1. Theoretical Modeling Methods

Theoretical modeling is fundamental to the investigation of heat and mass transfer mechanisms occurring during the drying of agricultural products. The core objective is to establish mathematical models that describe the temporal and spatial variations in the temperature and moisture content within the material. The drying models are generally classified into three categories: empirical, semi-empirical, and mechanistic (theoretical).

Empirical models are constructed through regression analysis of the experimental data, so as to establish relationships between the drying rate, moisture content, and time. Their application is simple, and they are widely usable; however, these models typically lack a physical basis and have limited ability to accurately capture the underlying heat and mass transfer mechanisms.

Semi-empirical models incorporate certain physical parameters, such as the effective moisture diffusion coefficient, into their empirical frameworks. This approach provides enhanced explanatory power and enables partial consideration of the physical processes governing drying, thereby bridging the gap between purely empirical and fully mechanistic approaches.

Mechanistic (theoretical) models are based on the fundamental principles of heat and mass conservation. By formulating coupled partial differential equations, typically involving Fick’s second law for mass transfer and energy balance equations for heat transfer, these models accurately characterize the coupled heat and mass transfer behaviors occurring during drying [87]. Advanced multiphase and multiphysics models further incorporate phenomena such as heat transfer at gas–solid interfaces, adsorption heat, and the latent heat of evaporation, thereby enabling a deeper understanding of the heat and mass transfer mechanisms acting in porous materials [88].

In agricultural-product drying studies, the modeling of plant tissue as a porous medium has become a mainstream approach. Porous-medium models can simultaneously represent heat transfer, moisture diffusion, and tissue deformation behaviors. Additionally, numerical solutions can provide detailed temperature and moisture-content distributions within the material. Such mechanistic modeling not only facilitates the prediction of the drying kinetics but also offers a theoretical foundation for optimization of the drying processes and equipment design.

### 6.2. Experimental Research Methods

Experimental research provides a foundational basis for obtaining heat and mass transfer parameters and validating theoretical models. Drying-kinetics experiments typically involve measurement of the temporal variation in the material moisture content under controlled drying conditions, with the drying curves then being analyzed to characterize the drying behavior. Such experiments enable the determination of key parameters, including the effective moisture diffusion coefficient and activation energy, and thus provide an empirical basis for model calibration and parameter estimation [89].

Modern experimental approaches increasingly employ advanced techniques to probe the internal heat and mass transfer mechanisms. Methods such as infrared thermography, magnetic resonance imaging (MRI), and low-field NMR (LF-NMR) can reveal temperature distributions and moisture migration pathways within the target material, thereby elucidating the internal transport processes occurring during drying [90]. In addition, the thermal properties of the material, such as its thermal conductivity and specific heat capacity, are determined through steady-state or transient experiments. These properties are often corrected to incorporate changes in the moisture content and serve as essential input parameters for theoretical and numerical models.

By integrating conventional drying-kinetics measurements with advanced imaging and thermal characterization techniques, experimental research facilitates a comprehensive understanding of the coupled heat and mass transfer processes, supports model validation, and provides guidance for optimizing drying conditions and equipment design.

### 6.3. Numerical Simulation and Computational Methods

With advances in computational capabilities, numerical simulations have become indispensable tools for the investigation of heat and mass transfer behaviors occurring during the drying of agricultural products. By discretizing and solving theoretical models, numerical methods can predict the spatiotemporal distributions of the temperature and moisture content of a given material, thereby providing an intuitive and quantitative basis for process optimization. Commonly employed numerical methods include the finite difference, finite element, and finite volume methods [87].

For drying systems involving complex geometries or multiphysics coupling, CFD is widely applied to simulate the coupled heat and mass transfer mechanisms between the drying medium and material, so as to capture interactions that are difficult to characterize experimentally [91]. In recent years, numerical simulations have been increasingly integrated with experimental data inversion for model-parameter calibration and improved prediction accuracy. Moreover, to address the limited adaptability of traditional models under complex operating conditions, and thus to predict and optimize drying processes, data-driven approaches (including machine learning techniques) have been introduced [92].

As reported in Table 3, each method for investigating the heat and mass transfer mechanisms acting during the drying of agricultural products has distinct advantages and limitations. Empirical models are simple and computationally light, but lack physical interpretation and are primarily suitable for rapid estimation. Semi-empirical models balance experimental data and theoretical explanations but require experimental validation of the parameters. Mechanistic models can accurately reflect the temperature and moisture distributions during drying; however, they are computationally intensive. Experimental methods, including drying kinetics and thermal property measurements, provide reliable parameters to underpin models; however, the experiments are time-consuming and highly condition-dependent. Microscopic transport-property measurements can reveal detailed local-heat and mass transfer mechanisms but require expensive instrumentation. Numerical simulations can predict drying performance under complex conditions and optimize processes, but require significant computational resources. Finally, data-driven methods rely on large datasets but are particularly suitable for industrial-scale applications and smart-agriculture scenarios.

In summary, theoretical modeling elucidates heat and mass transfer mechanisms, experimental research provides critical data to support these models, and numerical simulations enable process prediction and optimization. Synergistic integration of these approaches is essential to achieve a comprehensive understanding of the heat and mass transfer behaviors occurring during the drying of agricultural products.

## 7. Existing Issues and Development Trends

### 7.1. Major Existing Issues

Although considerable progress has been made in the study of the heat and mass transfer mechanisms involved in the drying of agricultural products, several critical issues remain. First, regarding the drying mechanisms, most existing models are based on simplified assumptions, such as the treatment of agricultural products as homogeneous porous media. As agricultural materials are heterogeneous and anisotropic, this assumption generates substantial discrepancies between the predicted moisture migration pathways and transfer rates and the actual behavior [87].

Second, model parameters are primarily determined from experimental measurements or empirical correlations. However, these parameters exhibit significant variability under different drying conditions. This variability is particularly pronounced in multi-field coupled drying processes, where the parameters change dynamically with the temperature, moisture content, and local structural conditions. Consequently, conventional models with fixed parameters often fail to describe the actual drying behavior [93].

Third, previous studies have predominantly focused on metrics such as the drying efficiency or product quality, with systematic investigations of the underlying heat and mass transfer mechanisms being neglected. As regards engineering applications, most research has been limited to laboratory or pilot-scale conditions. There is a lack of comprehensive studies examining the heat and mass transfer patterns arising under complex structural configurations, material stacking, and transient operating conditions typical of industrial-scale continuous-drying equipment. This gap significantly restricts the applicability and scalability of existing research findings [94].

### 7.2. Future Trends

In response to these challenges, future research in the field of agricultural-product drying is expected to progress in several key directions.

First, theoretical models will become increasingly refined and multi-scale, with the cellular-level water-migration mechanisms being integrated with macroscopic transport processes. Hence, the accuracy and predictive capability of the resultant drying models will be improved. In parallel, multiphysics coupling models, such as heat–mass–structure coupling models, are expected to receive greater attention, which will facilitate detailed characterization of the interactions among the material shrinkage, structural changes, and transport behavior [95].

Second, closer integration of experimental characterization and numerical simulation techniques can be expected. Advanced techniques, including NMR, CT, and IR thermography, can provide real-time three-dimensional mapping of temperature fields, moisture distributions, and structural changes during the drying process, thereby providing essential data for model validation and parameter inversion [96]. Simultaneously, high-performance computing and CFD will facilitate accurate simulation of heat and mass transfer behaviors in complex systems.

Third, technological innovations in drying processes will continue to be a major focus. A rational combination of methods such as hot-air, IR, MW, and vacuum drying will leverage the complementary advantages of different energy fields to yield enhanced drying efficiency, reduced energy consumption, and improved product quality.

Finally, the ongoing development of smart manufacturing and digital agriculture is promoting the adoption of data-driven drying modeling and control. The integration of machine learning with conventional heat and mass transfer models is enabling predictive monitoring, parameter optimization, and real-time process control. This approach will provide new strategies for the intelligent operation of drying systems as well as potential resolutions to the limitations of traditional models in the context of complex dynamic operating conditions.

### 7.3. Intelligent Drying Optimization, Digital Twins and Predictive Modeling

Intelligent drying optimization is becoming an important direction because the internal moisture state, product temperature and quality indicators change dynamically during drying. Traditional control strategies usually rely on fixed temperature, humidity and airflow settings, which are difficult to adapt to product heterogeneity and stage-specific transfer resistance. By integrating online sensors, moisture-content prediction, thermal imaging, NMR/near-infrared information and machine learning, drying systems can estimate the product state in real time and adjust process parameters accordingly.

Digital twins provide a promising framework for combining heat and mass transfer models with real-time process data. A drying digital twin can continuously update the predicted temperature field, moisture distribution, energy consumption and quality evolution, thereby supporting predictive control and multi-objective optimization. Future intelligent drying systems should not only minimize drying time, but also jointly optimize quality retention, specific energy consumption, carbon emissions and industrial throughput. Physics-informed machine learning and hybrid models that combine governing equations with data-driven correction are particularly valuable for improving model robustness under complex operating conditions [9,92].

## 8. Conclusions

The drying of agricultural products is fundamentally a transient and coupled heat and mass transfer process. Heat transfer provides the energy required for evaporation or sublimation, while moisture migration determines the drying rate, internal gradients and final product quality. This review analyzed agricultural-product drying from a mechanism-oriented perspective and compared typical convective, radiative, conductive and combined drying technologies in terms of their transfer pathway, efficiency, quality impact, energy use, carbon-footprint potential and industrial applicability.

Compared with previous reviews that mainly focus on single drying technologies, drying-kinetics models or product-quality indicators, this review compares different drying technologies from the perspective of heat and mass transfer, including the energy-input mode, moisture-migration pathway, dominant transfer resistance, quality effects, energy performance, carbon-footprint potential and industrial applicability. This mechanism-oriented framework helps clarify the advantages and limitations of different drying technologies and provides a reference for drying-process selection, combined drying system design and intelligent optimization control.

Conventional hot-air and belt drying remain the most mature and scalable technologies, but they are often limited by long drying time, internal diffusion resistance and high energy consumption. MW, RF and IR drying can intensify heat and mass transfer through volumetric or radiative heating, whereas vacuum and freeze drying can improve quality retention by reducing thermal and oxidative damage. However, each method has specific limitations, such as hot spots, limited penetration depth, high equipment cost or long process cycles. Combined drying offers a promising way to coordinate internal heating and external vapor removal, but requires more advanced process control.

From a methodological perspective, porous-medium-based models, governing heat and mass transfer equations, experimental characterization and numerical simulation together provide the theoretical and technical basis for understanding drying behavior. Nevertheless, current models still face challenges in describing heterogeneous and anisotropic plant tissues, dynamically changing transport parameters, shrinkage, microstructure evolution and industrial-scale continuous drying conditions.

Future research should focus on refined multi-scale and multi-field coupled models, quantitative comparison of drying performance and product quality, and stronger integration of online sensing, numerical simulation, digital twins and data-driven predictive control. In addition, energy efficiency, carbon footprint and industrial feasibility should be treated as key evaluation criteria together with drying kinetics and quality retention. Such a comprehensive framework will support the development of efficient, low-carbon and quality-preserving drying technologies for agricultural products.

## Figures and Tables

**Table 1:** Comparison of heat and mass transfer characteristics of agricultural products under different drying technologies.

Drying Technology	Main Heat Transfer Mechanism	Main Mass Transfer Mechanism	Heat & Mass Transfer Characteristics	Drying Efficiency & Energy	Applicability & Limitations	Representative References
Solar	Radiation + natural convection	Mainly surface evaporation	Unstable heat input; strongly affected by environmental factors	Low energy consumption; slow drying	Suitable for low-cost operations; limited control	[22,25]
Hot-air	Forced convection	Surface evaporation + internal diffusion	Long heat transfer path; controllable	Long drying time; high energy	Broad applicability; product quality may decrease	[28,30]
Belt	Convective heat transfer	Multi-layer diffusion + surface evaporation	Uneven airflow; stepwise temperature & moisture gradients	Continuous, high efficiency; higher energy	Suitable for large-scale production; uniformity may be an issue	[34,35]
Desiccant-wheel	Convection (low-humidity air)	Internal diffusion + surface evaporation	Low temperature; enhanced heat transfer	High efficiency; energy-saving	Complex system; high investment	[39,40]
MW	Electromagnetic heat transfer	Pressure-driven internal water movement	Direct internal heating; short path	Fast; high efficiency	Risk of overheating; potentially uneven heating	[41,43]
RF	Deep-penetration heating	Internal water migration	Large penetration depth; more uniform heating	High efficiency; lower energy	Suitable for thick layers; high equipment cost	[46,48]
IR	Radiation heat transfer	Surface evaporation + internal diffusion	Rapid surface heating; moderate internal	High initial drying rate; energy-efficient	Limited penetration; potential surface overheating	[55,56]
Vacuum	Conduction + low-pressure evaporation	Internal diffusion	Low temperature; reduced water boiling point	High drying efficiency; process-dependent energy consumption	High cost; suitable for heat-sensitive materials	[60,64]
Freeze	Sublimation at low temperature & vacuum	Solid water-phase change	No liquid phase; good structure retention	Superior quality; high energy consumption and long cycle	For high-value products only	[66,68]
Combined	Multiple modes combined	Multiple mechanisms	Coordinated internal/external heat & mass transfer	High efficiency; energy-saving	Complex system; high control requirements	[70,71]

**Table 2:** Industrial feasibility, energy efficiency and carbon-footprint considerations for different drying technologies.

Drying Technology	Industrial Feasibility	Energy-Efficiency Tendency	Carbon-Footprint Consideration	Main Bottleneck	Representative References
Solar	Low-cost small-scale application; limited industrial stability	Low auxiliary energy but unstable drying rate	Low direct emissions if auxiliary energy is minimized	Weather dependence and weak controllability	[22,25]
Hot-air/belt	High maturity and scalability	Often high specific energy consumption due to long drying time	Carbon footprint depends strongly on heat source and exhaust heat recovery	Internal diffusion resistance and heat loss	[30,33]
Desiccant-wheel	Moderate to high feasibility for heat-sensitive products	Potentially improved by latent/sensible heat recovery	Lower footprint possible when regeneration heat is recovered or renewable energy is used	Regeneration energy and system cost	[39,40]
MW/RF	Medium feasibility; suitable for high-value or specific product forms	Fast internal heating may reduce process time	Footprint depends on electricity source and field uniformity	Hot spots, non-uniform heating and equipment cost	[41,46]
IR	Good feasibility for thin-layer products and combined systems	High initial heating efficiency for surface dehydration	Potentially lower if combined with efficient airflow control	Limited penetration depth	[55,56]
Vacuum/ freeze	Feasible mainly for high-value products	Vacuum drying is process-dependent; freeze drying generally consumes high energy	Generally high unless renewable electricity and heat recovery are used	Capital cost, sealing requirements and long cycle	[60,66]
Combined	Promising for balancing product quality and energy use	Potentially lower specific energy consumption through staged/multi-field coupling	Potentially lower if optimized by multi-objective control	Complex parameter coordination and control	[70,71]

**Table 3:** Comparison of heat and mass transfer research methods.

Method Type	Research Object	Advantages	Limitations	Representative References
Empirical model	Moisture-content variation with drying time	Simple and computationally efficient	Lacks physical significance	[11,89]
Semi-empirical model	Moisture content + partial physical parameters	Good interpretability and applicability	Parameters rely heavily on experiments	[5,89]
Mechanistic model	Coupled temperature field and moisture field	Accurately reflects heat and mass transfer mechanisms	Complex model with high computational cost	[87,91]
Drying-kinetics experiment	Mass-loss variation during drying	Reliable and realistic parameters	Limited measurements; time-consuming	[25,30]
Local-transfer characteristic measurement	Temperature field and moisture migration	Suitable for analyzing microscopic mechanisms	Expensive instruments required	[85,90]
Thermophysical property experiment	Thermal conductivity, specific heat capacity	Provides input parameters for models	Strong dependence on experimental conditions	[13,91]
Numerical simulation	Temperature and moisture fields	Predicts complex drying conditions	Requires high-performance computation	[56,91]
Data-driven method	Heat and mass transfer prediction	Useful for process optimization	Strong dependence on data quality and quantity	[5,92]

## Data Availability

No new data were created or analyzed in this study. Data sharing is not applicable to this article.
